# Genetically elevated gamma-glutamyltransferase and Alzheimer's disease

**DOI:** 10.1016/j.exger.2018.03.001

**Published:** 2018-06

**Authors:** Setor K. Kunutsor, Jari A. Laukkanen, Stephen Burgess

**Affiliations:** a Translational Health Sciences, Bristol Medical School, University of Bristol, Southmead Hospital, Learning & Research Building (Level 1), Bristol, UK; b National Institute for Health Research Bristol Biomedical Research Centre, University of Bristol, Bristol, UK; c Institute of Public Health and Clinical Nutrition, University of Eastern Finland, Kuopio, Finland; d Faculty of Sport and Health Sciences, University of Jyväskylä, Jyväskylä, Finland; e Central Finland Health Care District Hospital, Jyväskylä, Finland; f MRC Biostatistics Unit, University of Cambridge, Cambridge, Cambridgeshire CB2 0SR, UK; g Cardiovascular Epidemiology Unit, University of Cambridge, Cambridge, Cambridgeshire CB1 8RN, UK

**Keywords:** AD, Alzheimer's disease, CI, confidence interval, GGT, gamma-glutamyltransferase, GRS, genetic risk score, GWAS, genome-wide association studies, IGAP, International Genomics of Alzheimer's Project, MR, Mendelian randomization, NHGRI, National Human Genome Research Institute, OR, odds ratio, SD, standard deviation, SNP, single nucleotide polymorphism, Gamma-glutamyltransferase, Alzheimer's disease, Mendelian randomization

## Abstract

Observational epidemiological evidence supports a linear and independent association between serum gamma-glutamyltransferase (GGT) concentrations and the risk of Alzheimer's disease (AD). However, the causality of this association has not been previously investigated. We sought to assess the causal nature of this association using a Mendelian randomization (MR) approach. Using inverse-variance weighted MR analysis, we assessed the association between GGT and AD using summary statistics for single nucleotide polymorphism (SNP)-AD associations obtained from the International Genomics of Alzheimer's Project of 17,008 individuals with AD and 37,154 controls. We used 26 SNPs significantly associated with GGT in a previous genome-wide association study on liver enzymes as instruments. Sensitivity analyses to account for potential genetic pleiotropy included MR-Egger and weighted median MR.

The odds ratio of AD was 1.09 (95% confidence interval, 0.98 to 1.22; *p*  *=*  0.10) per one standard deviation genetically elevated GGT based on all 26 SNPs. The results were similar in both MR-Egger and weighted median MR methods. Overall, our findings cannot confirm a strong causal effect of GGT on AD risk. Further MR investigations using individual-level data are warranted to confirm or rule out causality.

## Highlights

• The causal relevance of elevated GGT concentrations with increased AD risk was assessed.
• Genetically elevated serum GGT might not be causally related to AD.
• The associations observed in observational studies may be driven by biases.

## 1 Introduction

The global prevalence of Alzheimer's disease (AD) is expected to increase to 106 million in 2050 from 30 million in 2010 (Brookmeyer et al., 2007), which will further impose a significant economic burden on health systems and society as a whole (Brookmeyer et al., 2007). It has been reported that one-third of AD cases globally may be attributable to modifiable risk factors such as type 2 diabetes, hypertension, obesity, dyslipidemia, smoking, physical inactivity, smoking, and depression (Peters et al., 2008). Though these factors explain a large proportion of the risk of AD, its pathogenesis is still not fully established as the causal effects of these factors on AD risk are uncertain and other potential risk factors appear to be involved in AD development. There is therefore a need to critically evaluate putative risk factors that may have causal significance and help prioritize targets for effective prevention and management. Serum gamma-glutamyltransferase (GGT), a marker of liver injury and excessive alcohol consumption and also known to have pro-oxidant and pro-inflammatory properties (Emdin et al., 2005); has been linked with an increased risk of several chronic disease outcomes, including vascular and non-vascular conditions (Kunutsor et al., 2014a; Kunutsor, 2016; Kunutsor et al., 2015a; Kunutsor et al., 2015c; Kunutsor et al., 2014c; Kunutsor and Laukkanen, 2016).

In a recent assessment of the prospective association of baseline and long-term values of GGT with risk of dementia as well as AD in a population-based cohort of middle-aged to older Finnish men, we have demonstrated independent and log-linear associations between GGT and both outcomes (Kunutsor and Laukkanen, 2016). We reported multivariate adjusted hazard ratios of 1.33 and 1.24 for dementia and AD respectively per 1 standard deviation (SD) increase in baseline serum GGT. On correction for regression dilution, the corresponding estimates were respectively 1.51 and 1.37 per 1 SD increase in long-term serum GGT. In another recent longitudinal study conducted in older individuals, Praetorius Björk and Johansson demonstrated higher GGT concentrations to be associated with cognitive decline prior to death and vascular dementia in late life (Praetorius Bjork and Johansson, 2017).

It is however unclear whether the association between GGT and AD is free of unobserved confounding and/or reverse causation (Keavney, 2002; Petitti and Freedman, 2005). In the present study, we aimed to investigate whether the association between elevated GGT concentrations and increased AD risk is causal, using publicly available data of genome-wide association studies (GWAS) on liver enzymes and the International Genomics of Alzheimer's Project (IGAP), which is the largest genome-wide meta-analysis of AD reported to date (Lambert et al., 2013).

## 2 Methods

### 2.1 Single nucleotide polymorphisms associated with gamma-glutamyltransferase

Available GWASs reporting on the associations of SNP variants with serum GGT concentrations at genome-wide significant levels (*p* < 5 × 10^−8^) were included in the present analyses. Studies were identified by searching the original publications of GWASs for serum GGT that have been indexed by the National Human Genome Research Institute(NHGRI) GWAS catalogue (Hindorff et al., n.d.). The variants were identified from a large GWAS on liver enzymes comprising of 61,089 individuals from 21 European countries (Chambers et al., 2011). Genetic associations were reported as percentage changes in serum GGT concentrations. These were converted into changes in log-transformed GGT. We selected the 26 independent SNPs associated with serum GGT at genome-wide significance and which explained 1.9% of the total variation in serum GGT.

### 2.2 Data sources

For the present study, we used the publicly available GWAS data of the IGAP (Lambert et al., 2013), which is a large two-stage study based upon GWAS on individuals of European ancestry. In stage 1, IGAP used genotyped and imputed data on 7,055,881 single nucleotide polymorphisms (SNPs) to meta-analyse four previously-published GWAS datasets consisting of 17,008 Alzheimer's disease cases and 37,154 controls (The European Alzheimer's disease Initiative – EADI the Alzheimer Disease Genetics Consortium – ADGC The Cohorts for Heart and Aging Research in Genomic Epidemiology consortium – CHARGE The Genetic and Environmental Risk in AD consortium – GERAD). In stage 2, 11,632 SNPs were genotyped and tested for association in an independent set of 8572 Alzheimer's disease cases and 11,312 controls. Finally, a meta-analysis was performed combining results from stages 1 & 2. We selected the 26 GGT-related SNPs and extracted their effect estimates for AD (odds ratios, ORs) together with their accompanying standard errors from IGAP.

### 2.3 Power calculation

Estimates of power for the MR analysis on AD employed an online power calculator ([http://cnsgenomics.com/shiny/mRnd/]). We used the genetic sample size and case/control ratios together with the proportion of variance of GGT explained by the GRS. Using IGAP data, (Lambert et al., 2013) we had 82% power to detect a causal association with an OR of ≥1.20 per 1 standard deviation increase in serum GGT.

### 2.4 Statistical analyses

We used the reported SNP-GGT and SNP-AD association estimates to calculate a combined effect of the individual 26 SNPs on AD using an inverse-variance weighted (IVW) approach (Burgess et al., 2013). Sensitivity analyses involved calculating MR-Egger (Bowden et al., 2015) and weighted median (Bowden et al., 2016a) estimates, as the IVW estimates may be biased by inclusion of invalid genetic instrumental variables. MR-Egger regression analyses allowed the formal testing for the presence of directional pleiotropy. The combined effect of the SNPs is presented as the effect per 1 SD increase in genetically determined GGT with a 95% confidence interval (CI). A 1 SD increase was taken as a 0.65 unit increase in log-transformed GGT concentrations (Kunutsor and Laukkanen, 2016), equivalent to a 92% increase in GGT concentrations. The effect of GGT on AD from observational evidence was compared with the estimate obtained from the genetic analyses.

## 3 Results

### 3.1 Genetic variants for GGT and Alzheimer's disease

**Fig. 1:**
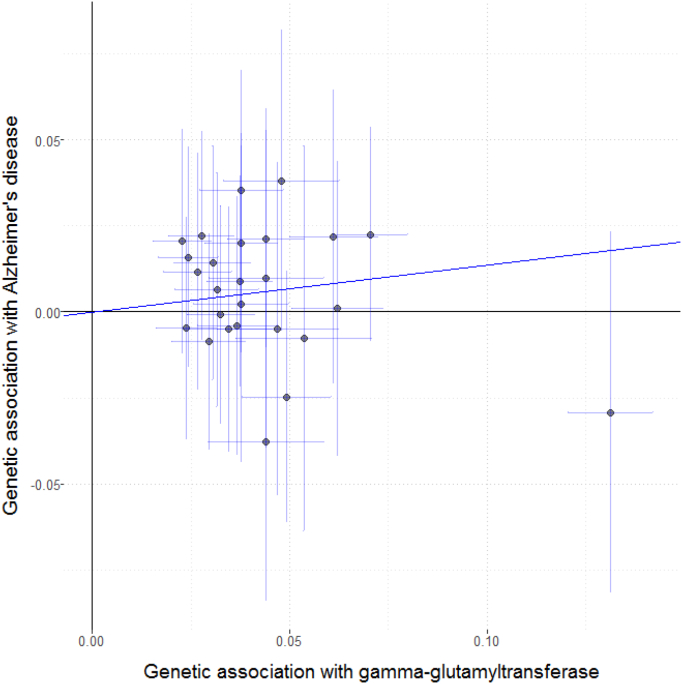
Associations of individual genetic variants with GGT and with Alzheimer's disease. Results displayed as the additive beta estimates and 95% confidence intervals. Horizontal axis presents the additive (per allele) associations of genetic variants with log-transformed GGT concentrations. Vertical axis presents the genetic associations with Alzheimer's disease risk (log odds ratio). The solid line represents the regression line of the inverse-variance weighted approach to combine the individual genetic variants; GGT, gamma-glutamyltransferase.

**Fig. 2:**
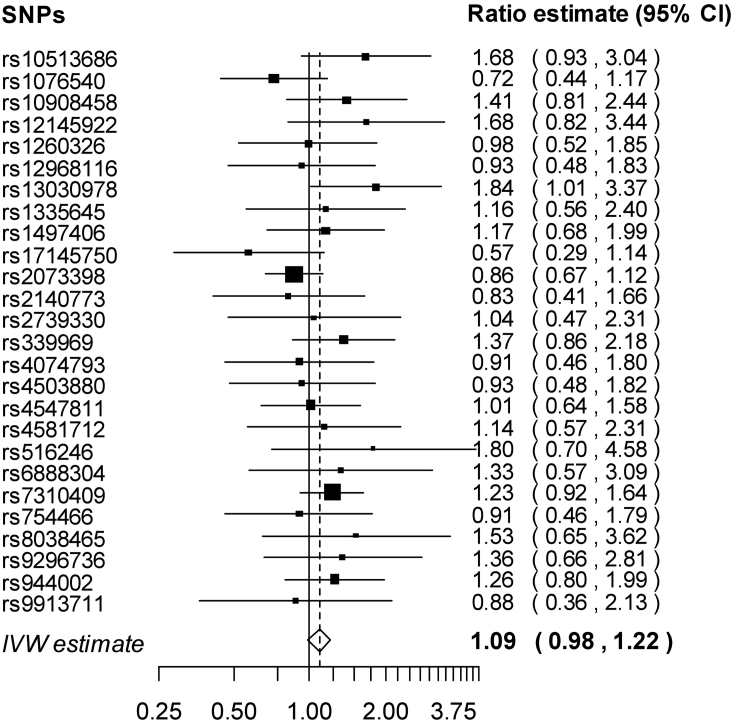
Causal estimates from individual SNPs and across all variants. Causal estimates and 95% confidence intervals (CI) based on each SNP in turn. Estimates represent odds ratios per 1 SD increase in GGT concentrations. The pooled estimate is from the inverse-variance weighted (IVW) method to combine evidence across the individual genetic variants; SNP, single-nucleotide polymorphism.

**Fig. 3:**
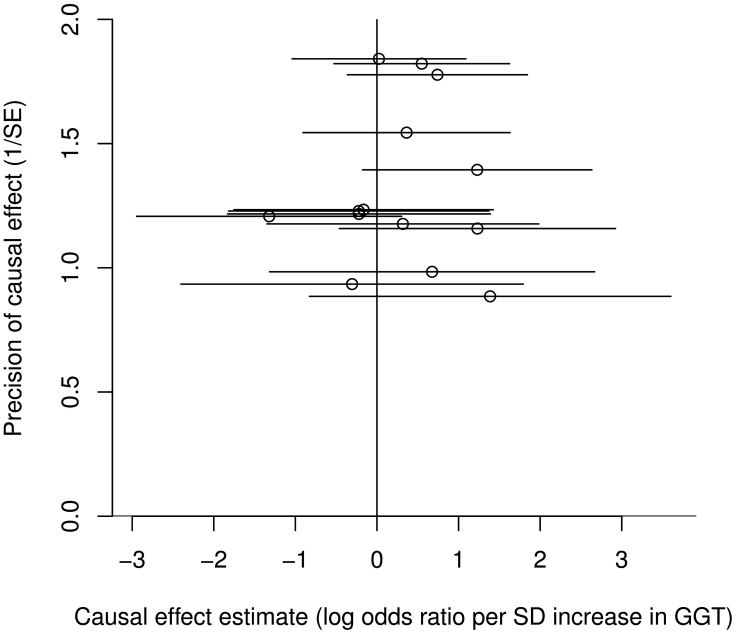
Funnel plot of genetic associations with GGT against causal effect estimates based on each genetic variant individually. GGT, gamma-glutamyltransferase.

Fig. 1 shows the associations of SNPs with GGT concentrations and AD risk. F-statistics of the individual SNPs ranged from 36.5 to 324.3 and the mean F-statistic of all 26 SNPs was 48.4. We found no evidence of an association between any of the individual SNPs for GGT and the risk of AD after accounting for multiple testing (only rs13030978 had *p* = 0.046, all other SNPs had *p* > 0.05). Using all the SNPs together in the IVW method, the causal OR for AD was estimated as 1.09 (95% CI, 0.98 to 1.22) per 1 SD increase in serum GGT concentration (Fig. 2). MR-Egger regression and weighted median estimators were consistent with IVW results, [OR 0.89 (0.69 to 1.16), *p* = 0.39] and [OR 1.06 (0.98 to 1.35), *p* = 0.092] respectively. Furthermore, in the MR-Egger regression analysis, there was no evidence of deviation of the intercept from zero [0.015, *p* = 0.070] (Table 1), indicating the absence of pleiotropy in the IVW SNPs and the I^2^ statistic was 91.7%, suggesting minimal bias (Bowden et al., 2016b). Fig. 3 shows a funnel plot of the genetic associations with GGT against the individual causal effect estimates for each variant. A visual inspection of the funnel plot suggests that there is little asymmetry present.

**Table 1:** Comparison between observational and genetically determined effect of gamma-glutamyltransferase on risk of Alzheimer's disease.

Exposure	Odds ratio (95% confidence)
Observational association per 1 SD higher GGT	
Baseline GGT	1.24 (1.01 to 1.53)
Long-term “usual levels” of GGT	1.37 (1.01 to 1.85)
Genetic estimate per 1 SD higher GGT	
Inverse-variance weighted method	1.09 (0.98 to 1.22)
MR-Egger regression method	0.89 (0.69 to 1.16)
Weighted median method	1.06 (0.98 to 1.35)

GGT, gamma-glutamyltransferase; MR, Mendelian randomization; SD, standard deviation; estimates are reported per 1 standard deviation increase in GGT.

## 4 Discussion

Given the previously observed association between elevated GGT concentrations and higher risk of AD, our aim within the present study was to investigate if the association was causal. Utilizing summary statistics of large-scale published GWAS, there was no evidence of an association between genetically determined serum GGT and risk of AD. These findings suggest that the observational association between GGT and AD might not be causal. Gamma-glutamyltransferase has been linked with the development of several vascular and non-vascular outcomes (Kunutsor et al., 2014a; Kunutsor et al., 2014b; Kunutsor et al., 2015c; Kunutsor et al., 2014c; Kunutsor et al., 2015b; Kunutsor et al., 2014d) and these relationships have been attributed to its pro-inflammatory and pro-oxidant properties (Emdin et al., 2005) as well as its direct involvement in atheromatous plaque formation (Franzini et al., 2009; Paolicchi et al., 2004). Gamma-glutamyltransferase may be linked to dementia or AD via these pathways (Kunutsor and Laukkanen, 2016), given the involvement of these processes in the development of these neurodegenerative conditions (Breteler, 2000; Gackowski et al., 2008; McGeer and McGeer, 2013; Yavuz et al., 2008; Zafrilla et al., 2006). Gamma-glutamyltransferase is also strongly related with metabolic abnormalities such as metabolic syndrome (Kunutsor et al., 2015b), obesity (Marchesini et al., 2005), and non-alcoholic fatty liver disease (Angulo, 2002), which are relevant to the development of AD (Kivimaki et al., 2017; Seo et al., 2016).

Given that the current results do not provide strong evidence for a causal association between GGT concentrations and AD risk, previous observational findings could have been the result of residual confounding and/reverse causality (elevated GGT concentrations being a consequence of the neurodegenerative process). However, the mechanisms postulated to link elevated GGT concentrations and increased AD are biologically plausible. The findings of a linear and independent association between GGT concentrations and AD risk in previous observational cohort studies are also suggestive of causality. Further studies are therefore needed to investigate this research question.

There are several strengths and weaknesses of the current investigation which merit consideration. Obviously, the current findings are important and less prone to bias than findings from traditional observational epidemiological studies, because causal investigations with the use of genetic variants are likely to be free from confounding, not subject to reverse-causation, and genetically-elevated serum GGT concentration is indicative of life-long values. The main data source for this study is the summary statistics from IGAP, which is the largest GWAS of AD reported to date (Lambert et al., 2013). Our MR approach employed the use of summarized publicly available data which precluded the ability to fully assess instrumental variable assumptions such as: (i) adequately address population stratification; (ii) test for the attenuation of genetic associations with the outcome on adjustment for the exposure of interest; (iii) test for assumptions required by instrumental variable methods for effect estimation such as pleiotropy (Burgess et al., 2013). Also, it was not possible to evaluate the biology of the GGT variants in detail because of the use of summary statistics from published studies; therefore there is a possibility that other biological pathways might explain the associations of some of these variants with GGT. However, it is unlikely that our results were affected by potential pleiotropy given the results from our MR-Egger-regression analyses. Our use of multiple SNPs also minimized the risk of pleiotropy. Furthermore, the SNP variants selected as instruments were strongly associated with GGT concentrations and their F-statistics were >10. A limitation of the MR approach is the limited strength of the SNPs to explain considerable variation in GGT concentrations, which may have restricted statistical power. However, the use of a large number of genetic variants and IGAP data may have provided sufficient power to detect any causal association of GGT with AD. Finally, since all participants in IGAP and the GWAS of liver enzymes are of European descent, the current findings cannot be necessarily generalized to other ethnic groups.

## 5 Conclusion

Overall, our findings cannot confirm any causal effect of GGT on AD risk. Given the limitations of the present study, further MR investigations using individual-level data are warranted to confirm or rule out causality.
